# Supplementary material for: The Photocatalytic Conversion of Carbon Dioxide to Fuels Using Titanium Dioxide Nanosheets/Graphene Oxide Heterostructure as Photocatalyst
Source: Nanomaterials (Basel). 2023 Jan 12;13(2):320. doi: 10.3390/nano13020320 (PMC9860753; doi:10.3390/nano13020320)
Supplement: Supplementary file 1 [file nanomaterials-13-00320-s001.zip › nanomaterials-2165623-supplementary.pdf]

nanomaterials

MDPI

Supplementary Materials

The photocatalytic conversion of carbon dioxide to fuels using titanium dioxide nanosheets/graphene oxide heterostructure as photocatalyst

Apisit Karawek [1], Kittipad Kittipoom [1], Labhassiree Tansuthepverawongse [1], Nutkamol Kitjanukit [1], Wannisa Neamsung [1], Napat Lertthanaphol [1], Prowpatchara Chanthara [1], Sakhon Ratchahat [2], Poomiwat Phadungbut [1], Pattaraporn Kim-Lohsoontorn [3] and Sira Srinives [1,*]

[1] Nanocomposite Engineering Laboratory (NanoCEN), Department of Chemical Engineering, Faculty of Engineering, Mahidol University, Nakhon Pathom, 73170, Thailand.
[2] Department of Chemical Engineering, Faculty of Engineering, Mahidol University, Nakhon athom, 73170, Thailand.
[3] Center of Excellence on Catalysis and Catalytic Reaction Engineering, Department of Chemical Engineering, Faculty of Engineering, Chulalongkorn University, Bangkok, 10330, Thailand
* Correspondence: sira.sri@mahidol.ac.th

Citation: Karawek, A.; Kittipoom, K.; Tansuthepverawongse, L.; Kitjanukit, N.; Neamsung, W.; Lertthanaphol, N.; Chanthara, P.; Ratchahat, S.; Phadungbut, P.; Kim-Lohsoontorn, P.; et al. The Photocatalytic Conversion of Carbon Dioxide to Fuels Using Titanium Dioxide Nanosheets/Graphene Oxide Heterostructure as Photocatalyst. *Nanomaterials* **2023**, *13*, 320. https://doi.org/10.3390/nano13020320

Academic Editor: Rafael Camarillo

Received: 31 December 2022
Revised: 6 January 2023
Accepted: 9 January 2023
Published: 12 January 2023

Copyright: © 2023 by the authors. Submitted for possible open access publication under the terms and conditions of the Creative Commons Attribution (CC BY) license (https://creativecommons.org/licenses/by/4.0/).

This PDF file includes:

Figure S1. Particle size (a−j) and thickness (k−p) distribution histograms of the TNS, TNP and composites. S2
Table S1. Dimensions of the photocatalysts. S3
Figure S2. EDS mapping of atomic composition of the CTNSG (a) and CTNPG (b). S3
Figure S3. Quantitative results of EDS analysis of the samples. S4
Figure S4. The N_2 adsorption–desorption isotherms (a) and the pore−size distribution curves (b) of the GO, TNSG and TNPG. S4
Table S2. Physicochemical and optical properties of the samples. S4
Table S3. CO_2 photoreduction products obtained from various operations. S5
Figure S5. The FT−IR spectra (a), XRD spectra (b), SEM image (c) and particle size distribution (d) of the virgin and reused CTNSG. S6

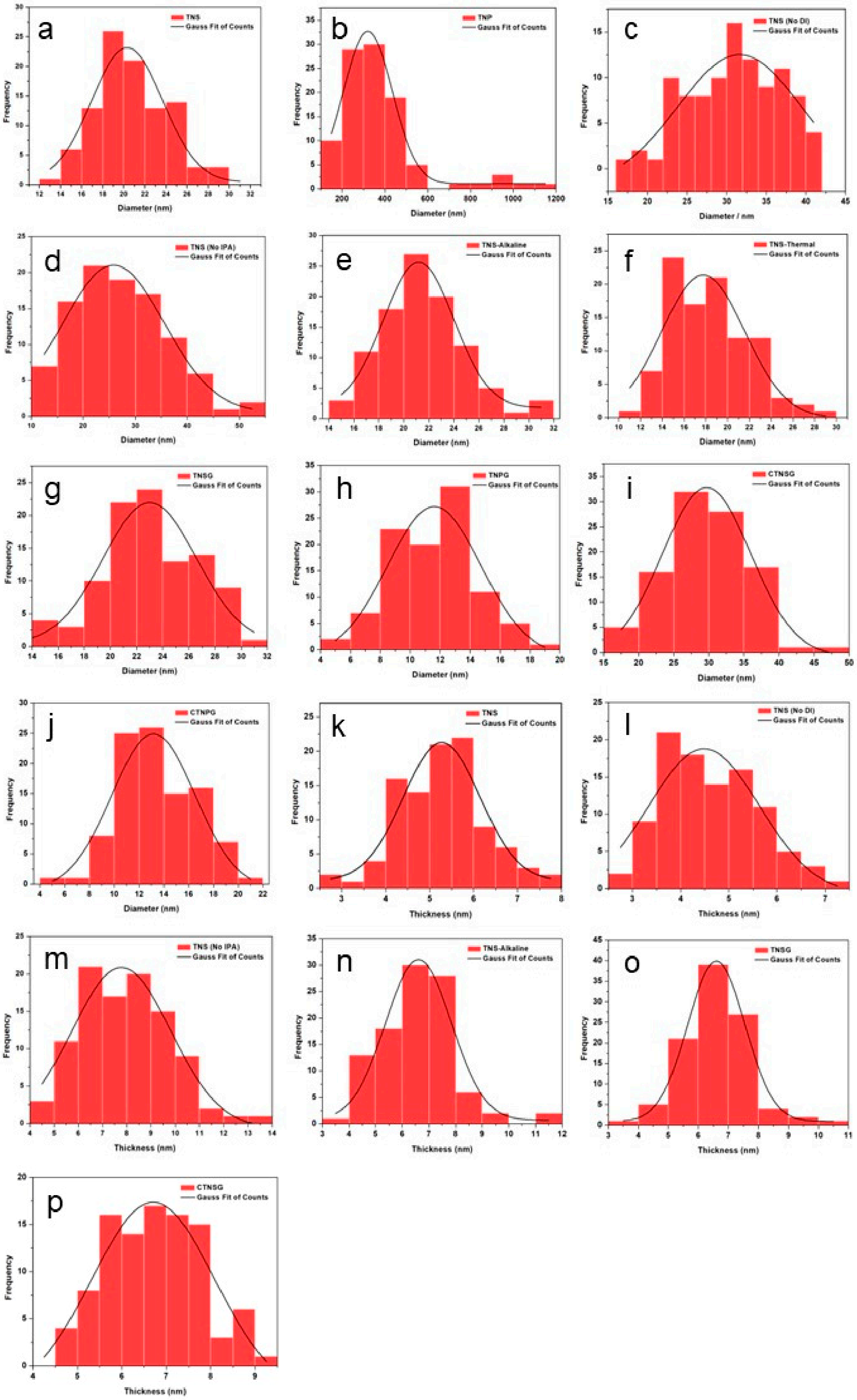

Figure S1. Particle size (a–j) and thickness (k–p) distribution histograms of the TNS, TNP and composites.

Table S1. Dimensions of the photocatalysts.

Catalyst	Width (nm)	Thickness (nm)
**TNS**	20.3 ± 0.4	5.3 ± 0.1
**TNS (HF+IPA)**	31.6 ± 0.8	4.5 ± 0.1
**TNS (DI+HF)**	25.8 ± 0.6	7.8 ± 0.2
**TNS-Alkaline**	21.2 ± 0.2	6.6 ± 0.1
**TNS-Thermal**	17.8 ± 0.6	-
**TNP**	320.6 ± 5.5	-
**TNSG**	23.0 ± 0.5	6.6 ± 0.1
**TNPG**	11.6 ± 0.5	-
**CTNSG**	29.7 ± 0.4	6.7 ± 0.1
**CTNPG**	13.2 ± 0.5	-

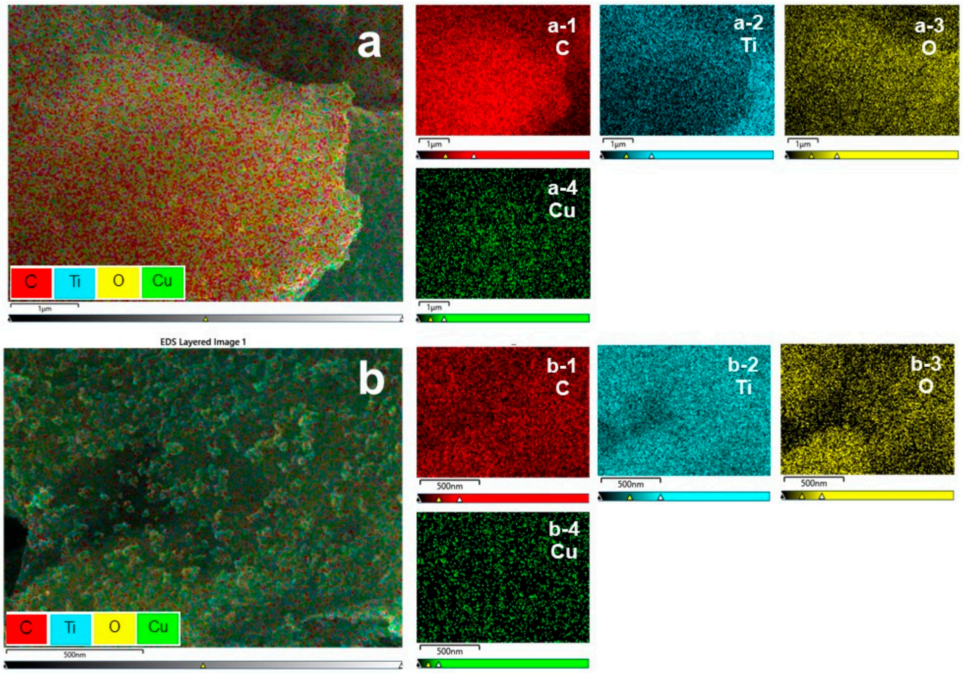

Figure S2. EDS mapping of atomic composition of the CTNSG (a) and CTNPG (b).

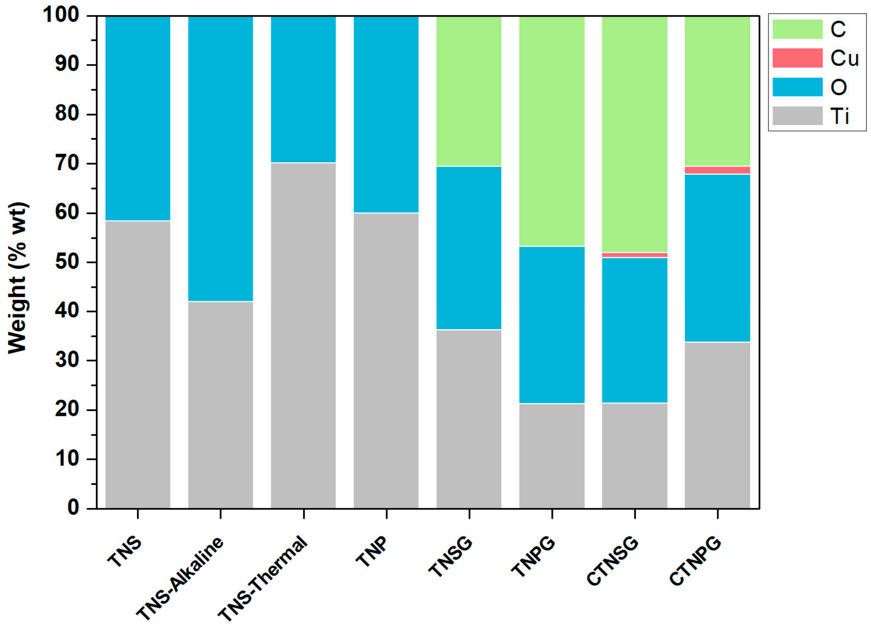

Figure S3. Quantitative EDS analysis of TNS, TNP and composites.

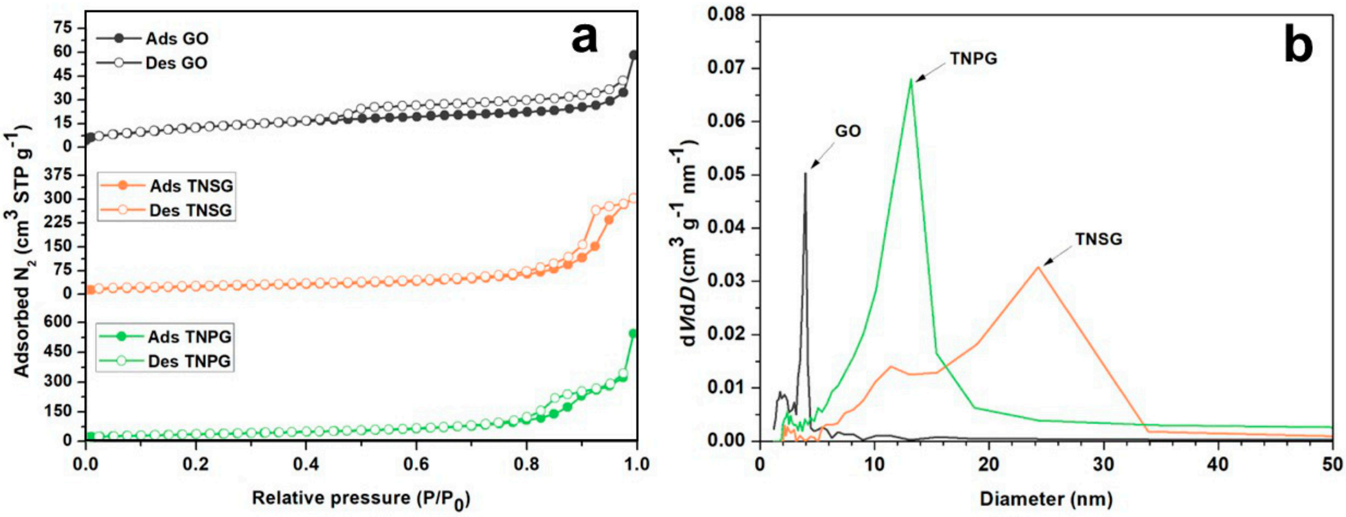

Figure S4. The N_2 adsorption–desorption isotherms (a) and the pore-size distribution curves (b) of the GO, TNSG and TNPG.

Table S2. Physicochemical and optical properties of the samples.

Catalyst	S_{BET} (m^2 g^{-1})	Average mesopore size 1 (nm)	Average mesopore size 2 (nm)	Band gap energy (eV)
**GO**	50.2	3.9	0	2.22
**TNS**	110.4	10.1	0	3.07
**TNP**	138.2	13.2	0	3.38
**TNSG**	86.4	11.4	24.29	2.98
**TNPG**	133.4	13.2	0	3.31
**CTNSG**	81.3	11.4	33.86	2.66
**CTNPG**	139.19	13.1	0	3.17

Table S3. CO_2 photoreduction products obtained from various operations.

**Catalyst**	**Condition[1]**		**Production rate (μmol $h^{-1}g_{cat}^{-1}$)**			**Total carbon consumption (μmol g_{cat}^{-1})**	**%PQE (x 10^{-2})**
	**Light source**	**%RH**	**Methane**	**Methanol**	**Acetone**		
**P25**	160 Watts	100	0.95	0	0.28	18.57	0.71
**TNS**	160 Watts	100	4.30	0	0.42	27.75	0.24
**TNS (HF+IPA)**	160 Watts	100	0.51	0	0.12	4.35	0.35
**TNS (DI+HF)**	160 Watts	100	1.85	0	0.56	17.65	1.39
**TNS-Alkaline**	160 Watts	100	4.65	0	0.48	30.45	2.63
**TNS-Thermal**	160 Watts	100	2.34	0	0.39	13.50	1.46
**TNP**	160 Watts	100	2.03	0	0.40	21.45	0.13
**TNSG**	160 Watts	100	2.81	0	0.69	24.40	0.20
**TNPG**	160 Watts	100	2.28	0	0.72	22.20	0.17
**CTNSG**	160 Watts	100	12.09	0	0.74	71.65	0.64
**CTNPG**	160 Watts	100	4.58	0	1.33	43.15	0.34
**CTNSG**	160 Watts	0	2.50	0	0.21	3.11	0.53
**CTNSG**	160 Watts	20	4.34	0	0.35	5.39	2.45
**CTNSG**	160 Watts	40	5.12	0	0.32	6.08	2.33
**CTNSG**	160 Watts	60	6.45	0	0.34	7.47	3.34
**CTNSG**	160 Watts	80	9.16	0	0.42	10.43	4.69
**CTNSG**	No	100	0	0	0	0	0.00
**CTNSG**	16 Watts	100	6.84	1.23	3.07	86.40	47.15
**CTNSG**	250 Watts	100	14.09	0	0.56	78.85	0.64
**CTNSG**	16 Watts / 160 Watts	100	6.35	1.16	2.91	81.2	44.83

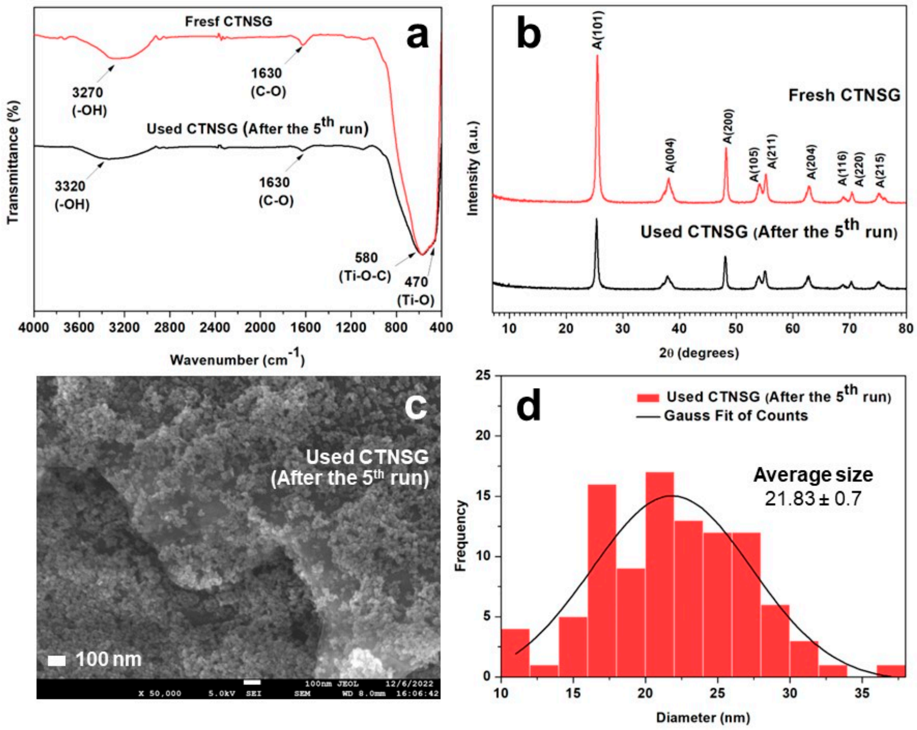

Figure S5. The FT−IR spectra (a), XRD spectra (b), SEM image (c) and particle size distribution (d) of the virgin and reused CTNSG.
